# Stationsersetzende Maßnahmen: neue Versorgungsperspektive für Österreich

**DOI:** 10.1007/s40211-022-00446-9

**Published:** 2022-11-07

**Authors:** Paul L. Plener, Patrick M. Frottier

**Affiliations:** 1 Universitätsklinik für Kinder- und Jugendpsychiatrie, Medizinische Universität Wien, Währingergürtel 18–20, 1090 Wien, Österreich; 2 Klinik für Kinder- und Jugendpsychiatrie und Psychotherapie, Universitätsklinik Ulm, Ulm, Deutschland; 3 Psychosozialer Dienst der Stadt Wien, Wien, Österreich

**Keywords:** Home-Treatment, Kinder- und Jugendpsychiatrie, Aufsuchende Behandlung, Multidisziplinäre Behandlung, Behandlungsform, Home-treatment, Child and adolescent psychiatry, Assertive care, Mutidisciplinary treatment, Treatment mode

## Abstract

Während intensive, aufsuchende Behandlungsformen in der Kinder- und Jugendpsychiatrie in vielen Ländern bereits erfolgreich evaluiert und etabliert wurden, ist die Entwicklung in diesem Bereich in Österreich am Beginn stehend. Im Zuge des sogenannten home-treatments erfolgt eine Behandlung von Patientinnen und Patienten, die aufgrund der Schwere ihrer Erkrankung ansonsten im stationären Setting behandelt werden würden durch ein multiprofessionelles Team im eigenen Haushalt. Das erlaubt unter anderem eine bessere Generalisierbarkeit therapeutischer Fortschritte und eine intensive Zusammenarbeit mit dem Familiensystem. Stationsersetzende Maßnahmen wie das home-treatment haben das Potenzial unabhängig von baulichen Strukturen eine intensive therapeutische Option darzustellen. Ein Ausbau dieser Behandlungsform basierend auf evaluierten Modellprojekten ist auch im österreichischen Gesundheitssystem wünschenswert.

## Abstract (en)

Although intensive, assertive treatment options in child and adoledcent psychiatry have been successfully evaluated and established in several countries, this development is just beginning in Austria. As part of the so called home-treatment, care is provided by a multiprofessional team to patients with severe mental disorders, who would otherwise be treated in an inpatiet setting at their own home. This opens up the opportunity for a better generalizability of therapeutic progress and an intensive work with the family system. Alternative models for inpatient care like home-treatment, show the potential for intensive therapeutic options independent of building structures. Further development of this therapeutic option based on evaluated model projects is desirable within the Austrian health care system.

## Einleitung

Die kinder- und jugendpsychiatrische Versorgungslandschaft Österreichs ist gekennzeichnet durch eine Unterversorgung in mehreren Bereichen. Neben dem Mangel an niedergelassenen Fachärztinnen und Fachärzten für Kinder- und Jugendpsychiatrie und Psychotherapeutische Medizin, sticht auch der Mangel an kinder- und jugendpsychiatrischen Behandlungsplätzen im stationären Rahmen ins Auge [1]. Auch wenn in den letzten Jahren ein Ausbau zu verzeichnen ist, so muss doch festgehalten werden, dass eine Zunahme an Bettenkapazitäten neben dem Zuwachs an Personal, auch einen Ausbau baulicher Infrastruktur bedeutet, was mitunter auch dazu führen kann, dass sich die Zeitperspektive verlängert, in der ebensolche Kapazitäten zur Verfügung gestellt werden können. Zudem bedeutet eine stationäre Behandlung auch eine Diskontinuität in den familiären und sozialen Beziehungen, die zwar in manchen Fällen durchaus auch zu einer gewollten Veränderung beitragen kann, in vielen Fällen aber als zusätzliche Belastung erlebt wird und mitunter auch die Schwelle zur Inanspruchnahme einer eigentlich notwendigen stationären Behandlung erhöht. Auch ist anzumerken, dass die Generalisierung von therapeutischen Erfolgen, die im stationären Setting erzielt werden, im häuslichen Rahmen nicht immer aufrechterhalten wird und zu Drehtüreffekten führen kann, wenn eine Symptomatik, die sich im stationären Rahmen gebessert hat, bei Rückkehr in das häusliche Setting exazerbiert.

## Definition der Versorgungsebene

In vielen Ländern existieren bereits seit einigen Jahren Behandlungskonzepte, die versuchen durch eine intensive Behandlung im häuslichen Rahmen ein der stationären Behandlung äquivalentes Therapieangebot zu schaffen [2]. Die Mehrzahl der Modelle zeichnet sich dadurch aus, dass sie von einer kinder- und jugendpsychiatrischen Klinik aus koordiniert werden, es findet sich auch in allen Modellen ein multiprofessionelles Team. Auffällig ist, dass die rechtlichen Grundlagen, sieht man von den neuen gesetzlichen Regelungen die sogenannte „Stationsäquivalente Behandlung“ (StäB) in Deutschland ab, in den meisten Ländern nicht eindeutig geklärt waren [3]. Seit dem 01.01.2017 wurde in der BRD mit dem „Gesetz zur Weiterentwicklung der Versorgung und der Vergütung für psychiatrische und psychosomatische Leistungen (PsychVVG)“ auch eine Abrechnungsrundlage geschaffen [4] und es findet sich ein deutlicher Ausbau dieser Behandlungsform. Bei Anwendung dieses Modells konnte auch im Langzeitverlauf gezeigt werde, dass die Eltern von Patientinnen und Patienten, die im Rahmen einer StäB behandelt wurden, nach etwas mehr als vier Jahren eine höhere Zufriedenheit mit der Behandlung berichteten als Eltern von Patientinnen und Patienten aus der Kontrollgruppe [5]. In Österreich ist diese Modell bislang nicht in der Regelversorgung angekommen, auch wenn durchaus ein Potenzial für die Implementierung dieser neuen Versorgungsform gesehen wird [3, 6].

## Bedarfsangaben

Im Bereich dieser stationsersetzenden Maßnahme existieren verschiedene Modelle, wobei häufig als Überbegriff der Terminus „home-treatment“ gebraucht wird. Im englischen Sprachraum existieren neben dem „home-treatment“ auch Begriffe wie „Assertive Community Treatment“ oder „Supportive Discharge Service“, die ebenfalls eine multiprofessionell ausgerichtete Betreuung im häuslichen Rahmen beschreiben [2]. Unter home-treatment soll in diesem Zusammenhang eine aufsuchende, intensive, multidisziplinäre Behandlung von psychisch erkrankten Kinder und Jugendlichen im häuslichen Umfeld verstanden werden. Das bedingt, das die im Krankenhaus arbeitende Berufsgruppen und die dargebotene Therapievielfalt, sich auch im home-treatment Setting wiederfinden muss, um vergleichbare Effekte erzielen zu können. Die Effektivität dieser Behandlungsmaßnahme ist mit stationären Aufenthalten vergleichbar [2] bei gleichzeitig leicht reduzierten Kosten im Gesundheitssystem [7, 8]. Dennoch erlaubt diese Behandlungsform durch das aufsuchende Herangehen und den Verzicht auf die Ressource „Bett“ frühzeitig einzugreifen und Chronifizierungen und daraus resultierende Folgekosten zu vermeiden. In diesem Zusammenhang können außerdem auch im Familiensystem befindliche, (noch) nicht klinisch auffällige Kinder und Jugendliche und/oder psychisch kranke Eltern gesehen werden, was ebenso dazu angetan ist, schnellere notwendige Behandlungen anbahnen zu können.

Prinzipiell sind mehrere Modelle für das home-treatment denkbar. So wurden von Shepperd und Kollegen [9], mehrere Formen der Alternativen zu stationären Behandlungen diskutiert, wie etwa: Multi-systemische Therapie (MST) im häuslichen Umfeld, spezialisierte, hochfrequente ambulante Angebote, intensives home-treatment und intensive häusliche Krisenintervention.

Fokussiert man auf die beiden letztgenannten Bedingungen, so können einerseits Modelle zur akuten Krisenintervention angewandt werden, wobei die Behandlung dann zeitlich begrenzt werden muss um die gesamte Falllast eingrenzen zu können. Daneben existiert ein Modell, wonach ein home-treatment Team für eine längere Behandlungsepisode (etwa 3–6 Monate) in einer Familie tätig wird. Auch hier gilt es, das komplette Angebot einer multidisziplinären Behandlung vergleichbar dem stationären Bereich vorzuhalten.

## Strukturqualitätskriterien/Mindeststandards

Von Zechmeister-Koss und Kollegen [3] wurden basierend auf der Analyse von sechs unterschiedlichen home-treatment Modellen in verschiedenen Ländern auf den Aspekt der Multiprofessionalität des Teams hingewiesen. Dieses setzt sich in den meisten Projekten aus Ärztinnen/Ärzten für Kinder- und Jugendpsychiatrie, Psychologinnen/Psychologen, Sozialarbeiterinnen/Sozialarbeiter, sowie administrativem Personal zusammen [3]. Im Rahmen der StäB wurden Kriterien für die Durchführung definiert (s. Tab. 1).

**Table 1:** 

Behandlung durch multiprofessionelles Team (min. drei Berufsgruppen im Team oder im Krankenhaus) unter Leitung von Fachärztin/Facharzt für Kinder- und Jugendpsychiatrie
Einverständnis aller Personen im Haushalt > 18 Jahre
Min. eine wöchentliche Visite mit Patientinnen‑/Patientenkontakt mit Fachärztinnen‑/Facharztstandard
Min, eine wöchentliche multiprofessionelle Fallbesprechung
Behandlung auf Grundlage von individuellem Therapieplan
Min. ein direkter Patientinnen‑/Patientenkontakt durch min. ein Teammitglied/Tag
Erreichbarkeit min. eines Teammitglieds werktags in den Tagdienstzeiten; 24/7 Erreichbarkeit des Krankenhauses
Option zur vollstationären Aufnahme bei kurzfristiger Verschlechterung
Kontakte können verschiedener Art sein (ärztliche oder psychologische Einzelgespräche, Familiengespräche, Beratung, Psychotherapie, Entspannunsgverfahren, Helferkonferenzen, Psychoeduaktion, Elterntraining, alltagsbezogenes Training, Ergotherapie, Bewegunstherapie, …).

In Österreich fand die aufsuchende Behandlung aus dem Krankenhaus heraus, bisher im Rahmen eines Projekts der Klinischen Abteilung für Kinder- und Jugendpsychiatrie und Psychotherapie des Universitätsklinikums Tullns statt. Andere Projekte sind den Autoren nicht bekannt. Vor dem Hintergrund des Auftrags die multiprofessionelle Behandlung im stationären Setting im Rahmen eines stationsersetzenden Modellprojekts abzubilden, wurde zum 01.03.2021 ein home-treatment Projekt in Wien durch die Projektpartner Psychosozialer Dienst der Stadt Wien und Universitätsklinik für Kinder- und Jugendpsychiatrie an der Medizinischen Universität Wien initiiert. Damit soll der Forderung das home-treatment im österreichischen Gesundheitssystems zu erproben und diesen Vorgang zu evaluieren [3] Rechnung getragen werden. Bislang existieren im Rahmen des Projekts zwei Teams, die für je 5–7 Patientinnen und Patienten zuständig sind, wobei bereits jetzt parallel weitere Teams aufgebaut werden. Die Teams werden fachlich fachärztlich kinder- und jugendpsychiatrisch geleitet, wobei die Teams von einer Teamleitung aus dem Bereich soziale Arbeit/Sozialpädagogik koordiniert werden. Das Personal dieser Teams setzt sich aus mehreren Berufsgruppen zusammen: Kinder- und Jugendpsychiaterin/Kinder- und Jugendpsychiater, Psychologin/Psychologe, Pflegekraft, Sozialarbeiterin/Sozialarbeiter, Sozialpädagogin/Sozialpädagoge und Ergotherapeutin/Ergotherapeut, wobei die Teamzusammensetzungen hinsichtlich der Stellenanteile variieren können.

## Umsetzungsempfehlung, Zukunftsideen

Von Zechmeister-Koss und Kollegen [3] wurde darauf hingewiesen, dass aus Sicht der Implementierungsforschung home-treatment-Projekte als komplexe Interventionen zu betrachten sind, da es viele interagierende Komponenten gibt, der Schweregrad der psychischen Erkrankung der zu behandelnden Patienten relativ hoch ist, ein multidisziplinäres Team auf mehreren Organisationsebenen mitarbeiten muss und die Durchführung des home-treatments einen hohen Grad an Flexibilität oder auch Individualisierung benötigt. Das trägt auch dazu bei, dass die Vergleichbarkeit verschiedener Interventionen über Ländergrenzen hinweg erschwert ist. Umso mehr ist es wichtig, dass ausgehend von Best-Practice-Modellen im internationalen Kontext eine Adaptierung an die österreichische Versorgungsituation stattfindet. In einer aktuellen Befragung zu Implementierungsaspekte anhand eines Fragebogens, der von österreichischen Fachkräften in der Kinder- und Jugendpsychiatrie beantwortet wurde, wurden mehrere Herausforderungen in der Implementierung für Hometreatment in Österreich identifiziert [3]. Hier wurde die Anbindung an einen Krankenhauskontext als günstig für die Implementierung eines home-treatments gesehen, wobei die Anbindung hier vor allem an den ambulanten Bereich stattfinden kann oder aber auch eine andere Möglichkeit in der Anbindung an kinder- und jugendpsychiatrische Ambulatorien mit Tagesklinik, auch im Sinne einer tagesklinisch-äquivalenten Behandlung (TäB), gesehen wird. Auch hinsichtlich der geografischen Lage zeigen sich Herausforderungen, da das Aufsuchen der Familien nur in einem bestimmten Radius möglich ist, da ansonsten die Fahrzeiten des Teams zu viele Ressourcen benötigen. Hier könnte die Anbindung an dislozierte ambulante Einrichtungen Potenzial haben um auch rurale Gegenden besser versorgen zu können. Das Familiensystem rückt bei diese Behandlungsform noch stärker in das Zentrum der Behandlung. Das birgt einerseits eine Umstellung in sich im Vergleich zu den gewohnten Formen der kinder- und jugendpsychiatrischen Arbeit, andererseits ergibt sich auch das Potenzial, dass Schwierigkeiten, die in der Familie entstehen auch direkt identifiziert und adressiert werden können. Hierbei wurde auch darauf verwiesen, dass die Bereitschaft der Familienangehörigen, ebenso wie die Bereitschaft der Patientinnen und Patienten, wesentlich zum Erfolg des Hometreatments beiträgt, und der Abschätzung dieser Bereitschaft zu Beginn der Behandlung kommt ein hoher Stellenwert zu. Hinsichtlich der rechtlichen Fragestellungen muss besonders darauf geachtet werden, dass Mitarbeitende von Krankenanstalten nicht immer die Möglichkeit haben außerhalb der Krankenanstalt in ihren Professionen tätig zu werden. Dies muss vor Beginn eines home-treatment-Programmes vertraglich geklärt werden um auch eine Rechtssicherheit für Mitarbeitende zu schaffen.

Auch wenn sich diese Herausforderungen stellen, so ist das Potenzial eines Ausbaus stationsersetzender Behandlungen dennoch groß. Die Generalisierbarkeit neu erworbener Fähigkeiten im Alltag wird einer kontinuierlichen Erprobung ausgesetzt, auch das Training neuer Fertigkeiten kann in vivo und meist mit wesentlich geringerem Planungsaufwand als im stationären Setting erfolgen. Die Auseinandersetzung mit den Dynamiken des familiären Systems ist unausweichlich, was auch dazu führt, dass durch die Anwesenheit im familiären Alltag viele Dinge schneller „auf den Punkt“ gebracht werden können, da unmittelbar Erlebtes aufgegriffen werden kann. Gerade vor dem Hintergrund, dass die Bettenmessziffern laut dem Österreichischen Strukturplan Gesundheit nicht erreicht werden (wobei es hier große Länderunterschiede gibt), lässt sich eine home-treatment Intervention auch losgelöst von baulichen stationären Strukturen (allerdings mit der Möglichkeit einer stationären Behandlungsmöglichkeit im Krisenfall in der Hinterhand) betreiben, ohne auf einen multiprofessionellen Ansatz in der Behandlung verzichten zu müssen. Es wäre daher wünschenswert, wenn aus evaluierten Modellprojekten eine neue Versorgungsform etabliert werden könnte, die bundesweit regional zur Verfügung steht.

## Interessenkonflikt

P.L. Plener und P.M. Frottier geben an, dass kein Interessenkonflikt besteht. P.L. Plener: In den letzten fünf Jahren erhielt er Forschungsförderung durch das Bundesministerium für Bildung und Forschung (BMBF), das Bundesinstitut für Arzneimittel und Medizinprodukte (BfARM), die Baden-Würrtemberg Stiftung, die EWSA, den Fonds zur Förderung wissenschaftlicher Forschung (FWF), den Hochschuljubiläumsfonds, den Zukunftsfonds und den Nationalfonds. Er war als Vortragender für GSK, Janssen, Infectopharm und Oral B tätig. Er ist als Berater für Delta4 und Boehringer-Ingelheim tätig.
